# Proteomic changes in the milk of water buffaloes (*Bubalus bubalis*) with subclinical mastitis due to intramammary infection by *Staphylococcus aureus* and by non-aureus staphylococci

**DOI:** 10.1038/s41598-019-52063-2

**Published:** 2019-11-01

**Authors:** Salvatore Pisanu, Carla Cacciotto, Daniela Pagnozzi, Giulia Maria Grazia Puggioni, Sergio Uzzau, Paolo Ciaramella, Jacopo Guccione, Martina Penati, Claudia Pollera, Paolo Moroni, Valerio Bronzo, Maria Filippa Addis

**Affiliations:** 1 Porto Conte Ricerche, Alghero, Italy; 2 Dipartimento di Scienze Biomediche, Università degli Studi di Sassari, Sassari, Italy; 3 Dipartimento di Medicina Veterinaria e Produzioni Animali, Università di Napoli Federico II, Naples, Italy; 4 Dipartimento di Medicina Veterinaria, Università degli Studi di Milano, Milan, Italy; 5 Animal Health Diagnostic Center, Cornell University, Ithaca, NY USA; 6 Present Address: Dipartimento di Medicina Veterinaria, Università degli Studi di Sassari, Sassari, Italy

**Keywords:** Proteomics, Infectious-disease diagnostics

## Abstract

Subclinical mastitis by *Staphylococcus aureus* (SAU) and by non-aureus staphylococci (NAS) is a major issue in the water buffalo. To understand its impact on milk, 6 quarter samples with >3,000,000 cells/mL (3 SAU-positive and 3 NAS-positive) and 6 culture-negative quarter samples with <50,000 cells/mL were investigated by shotgun proteomics and label-free quantitation. A total of 1530 proteins were identified, of which 152 were significantly changed. SAU was more impacting, with 162 *vs* 127 differential proteins and higher abundance changes (P < 0.0005). The 119 increased proteins had mostly structural (n = 43, 28.29%) or innate immune defence functions (n = 39, 25.66%) and included vimentin, cathelicidins, histones, S100 and neutrophil granule proteins, haptoglobin, and lysozyme. The 33 decreased proteins were mainly involved in lipid metabolism (n = 13, 59.10%) and included butyrophilin, xanthine dehydrogenase/oxidase, and lipid biosynthetic enzymes. The same biological processes were significantly affected also upon STRING analysis. Cathelicidins were the most increased family, as confirmed by western immunoblotting, with a stronger reactivity in SAU mastitis. S100A8 and haptoglobin were also validated by western immunoblotting. In conclusion, we generated a detailed buffalo milk protein dataset and defined the changes occurring in SAU and NAS mastitis, with potential for improving detection (ProteomeXchange identifier PXD012355).

## Introduction

The water buffalo (*Bubalus bubalis*) is the second most important dairy species after the cow (*Bos taurus*)^1^. Approximately 15% of the world milk production is from buffaloes and the Asian continent, with a population of about 150 million animals, is the major producer. In Europe, Italy takes the lead with a population of about 400,000 heads (95% of the European population) for 200,000 tons of milk per year^2^. The reason for the increasing interest in buffalo breeding over recent years is the popularity of buffalo Mozzarella cheese (Protected Designation of Origin-P.D.O.) and almost lack of competition in the EU-area for this type of cheese. Mediterranean buffaloes are typically reared in central and southern Italy, and 80% of all Italian buffalo milk production originates from the Campania region. Mediterranean buffalo’s milk production is ranked 4^th^ in the Italian agricultural economy concerning sales volume in the entire country (more than 320 M€ and over 15,000 workforces)^3^.

Buffalo milk is a highly valuable product, being paid at least twice the price of bovine milk, and the European Community has not defined production quotas. Italian buffaloes produce small quantities of milk; the average production in a standard lactation cycle (approximately 270 d) is about 2,000 kg^3^, but milk is characterised by a higher percentage of total solids, including proteins, fat, and minerals, than cow’s milk. In view of its limited productions and high value, one of the costliest diseases is mastitis. Although buffaloes are traditionally considered less susceptible to mastitis than cattle^3,4^, some researchers have reported similar mastitis frequencies for the 2 species^5–8^, and the high prevalence of subclinical intramammary infections (IMI) might lead to underestimate the issue^5,9^. In dairy ruminants, the somatic cell count (SCC) is typically used as an inflammatory indicator to diagnose mastitis, as a proxy of the number of neutrophils in milk. Accordingly, the current classification defines as affected by subclinical mastitis all Mediterranean buffaloes without clinical signs having a SCC >200,000 cells/mL^3^.

The main bacterial species isolated from water buffalo milk are staphylococci. *Staphylococcus aureus* (SAU) is the most impacting intramammary pathogen^3,5,7,10^, but non-aureus staphylococci (NAS) are most frequently found; in our previous study, NAS were present in 78.4% of culture-positive samples^9^. Consequently, there is clearly a need to understand the impact of staphylococcal IMI on water buffalo milk productions and to improve its detection^3,10^. Proteomic investigations are a powerful means for assessing changes in milk proteins and for uncovering novel diagnostic markers. Specifically, shotgun proteomic analysis pipelines can provide a profound characterisation of milk proteins, highlighting the alterations introduced by IMI and identifying possible markers of an inflammatory condition^11–14^. However, little information is available in healthy and diseased buffalo milk. Sparse proteomic analyses, especially when compared to cow mastitis, have been performed on this species^15,16^. A recent proteomic investigation provided useful information on the profile of buffalo milk with mastitis, but it was limited to one-dimensional and two-dimensional electrophoresis of whey followed by the identification of the main protein spots for the purpose of setting up reference maps and of identifying acute phase proteins (APP)^17^. Here, we applied a shotgun proteomics workflow combining high performance orbitrap mass spectrometry with label-free quantitation to the milk of animals with subclinical mastitis due to staphylococcal IMI and of healthy animals with the following aims: to provide a vast dataset of buffalo milk proteins, to evaluate and understand the impact of subclinical staphylococcal mastitis on the buffalo milk proteome, to assess the differential impact of SAU and NAS IMI, and to identify novel markers for improving mastitis detection.

## Results

### Animals and milk samples

To assess the changes induced on the buffalo milk proteome by high-SCC subclinical mastitis due to staphylococcal IMI, 12 quarter milk samples were subjected to comparative proteomic analysis: 6 with SCC >3,000,000 cells/mL, of which three SAU-positive and three NAS-positive; and 6 with SCC <50,000 cells/mL, all culture-negative. SAU-positive and NAS-positive samples were collected from quarters positive for the California Mastitis Test (CMT) and classified as affected by subclinical mastitis, while all control quarters were CMT-negative and classified as healthy. The quarters belonged to 12 different animals. Sample characteristics are outlined in Table 1.

**Table 1:** Sample groups, milk samples, and their characteristics.

Sample group	SCCa (cells/mL × 10^3^)	Bacteriology
*Positive*	*Mean value* 5545 (*CMT*^*b*^ + 3)	
1	4091	*Staphylococcus aureus*
2	3782	*Staphylococcus aureus*
3	8440	*Staphylococcus aureus*
4	>10000	Non-aureus staphylococci
5	4035	Non-aureus staphylococci
6	2924	Non-aureus staphylococci
*Negative*	*Mean value* 23.5 (*CMT Neg*)	
7	46	Negative
8	37	Negative
9	30	Negative
10	14	Negative
11	8	Negative
12	6	Negative

^a^SCC: Somatic Cell Count. ^b^CMT: California Mastitis Test.

### SDS-PAGE patterns of *Staphylococcus*-positive and healthy control milk

**Figure 1:**
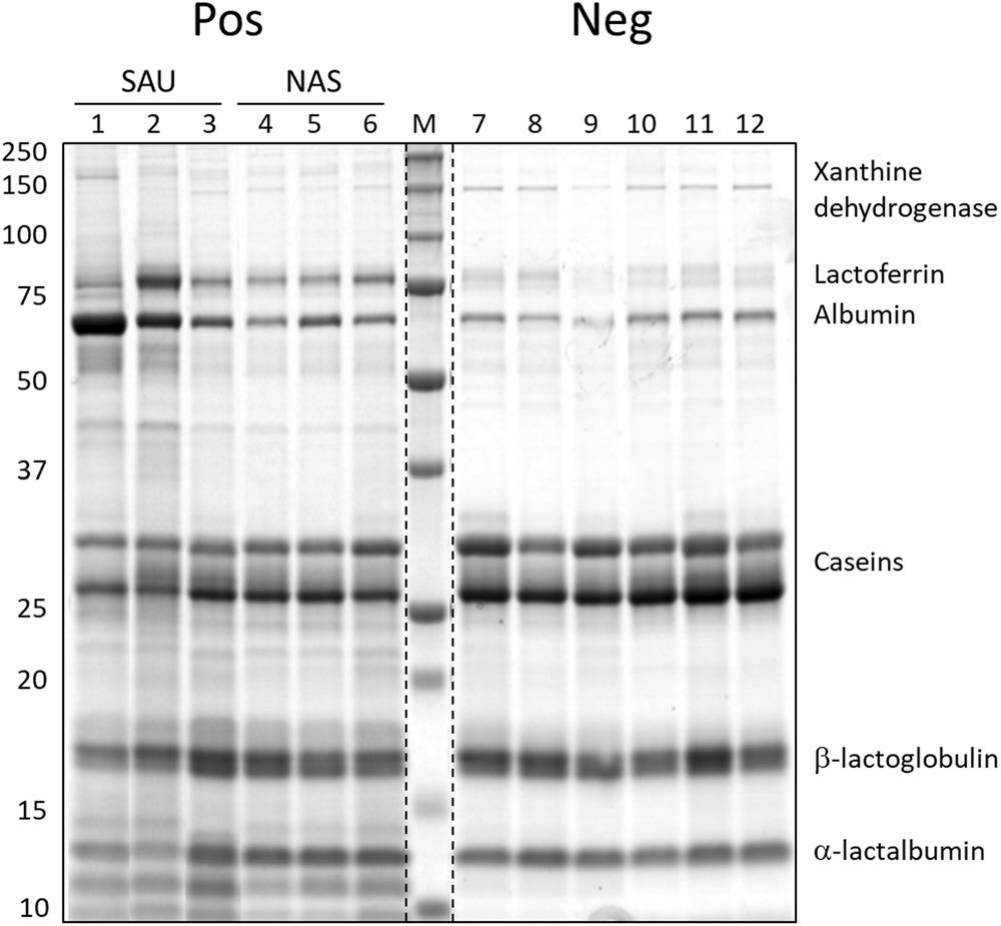
SDS-PAGE profiles of skim milk samples before trypsinisation for shotgun analysis. Pos: culture-positive samples. Neg: culture-negative samples. SAU: milk samples positive for *Staphylococcus aureus*. NAS: milk samples positive for non-aureus staphylococci. M: molecular weight markers. Sample numbers correspond to those listed in Table 1. Molecular weight references are indicated on the left. Proteins with a molecular weight corresponding to the main electrophoretic bands are indicated on the right as a reference. One microliter of skim milk was loaded in each lane.

The SDS-PAGE analysis carried out on solubilised skim milk proteins before trypsinisation for shotgun analysis anticipated the presence of several major changes related to staphylococcal IMI (Fig. 1). The major protein bands corresponding to lactoferrin, albumin, caseins, alpha-lactalbumin and beta-lactoglobulin were clearly affected^17^. Specifically, lactoferrin and albumin increased in staphylococcus-positive samples, while caseins, alpha-lactalbumin, and beta-lactoglobulin decreased. The appearance of other bands could also be observed, especially at low molecular weight. Alterations were generally more evident in SAU-positive milk (Fig. 1, lanes 1, 2, 3) than in NAS-positive milk (Fig. 1, lanes 4, 5, 6).

### Shotgun proteomics and differential analysis

A shotgun proteomic analysis combining filter-aided sample preparation (FASP), high performance reverse-phase chromatography, and high resolution orbitrap mass spectrometry was carried out to gain a more detailed picture of the alterations caused by staphylococcal infections on the water buffalo milk proteome. A total of 1530 proteins were identified, of which 1034 eligible for differential analysis (Supplementary File, Sheet 1). Mass spectrometry raw data have been deposited to the ProteomeXchange Consortium (http://proteomecentral.proteomexchange.org) via the PRIDE partner repository^18^ with the dataset identifier PXD012355. Differential protein abundances were assessed in: (i) all staphylococcus-positive vs control milk; (ii) only SAU-positive vs control milk; (iii) only NAS-positive vs control milk. Results are detailed in Supplementary File 1, Sheets 2–5, and are summarised in Table 2.

**Table 2:** Summary of differential proteomic results.

	Eligible for comparison*	Changed**	Differential*** 1.5 ≤ R_SC_ ≤ −1.5	Increased*** R_SC_ ≥ 1.5	Decreased*** R_SC_ ≤ −1.5
Posa/neg^b^	1034	302	152	119	33
SAU^c^/neg	1025	268	162	128	34
NAS^d^/neg	940	202	127	108	19

^a^Pos: Staphylococcus-positive. ^b^Neg: negative. ^c^SAU: only *Staphylococcus aureus*-positive. ^d^NAS: only non-aureus staphylococci-positive. *Proteins identified in at least two biological replicates and with ≥2 spectral counts in at least one sample of the experimental group. **p ≤ 0.05 by the beta-binomial test with FDR correction according to Benjamini-Hochberg. ***p ≤ 0.05 by the beta-binomial test with FDR correction according to Benjamini-Hochberg, and R_SC_ ≤ −1.5 or ≥1.5.

When considering all staphylococcus-positive *vs* healthy control milk, 302 proteins showed significant changes (p ≤ 0.05) in their relative spectral count (R_SC_). Of these, 152 passed also the selected abundance threshold (R_SC_ ≥ 1.5 or R_SC_ ≤ −1.5); 119 were increased and 33 were decreased in staphylococcal mastitis (differential proteins, Table 3). Of the 119 increased differential proteins, 63 were identified in all staphylococcus-positive milk samples with at least 2 peptide spectrum matches (PSMs) and were never detected in healthy milk (Table 3, asterisk). When considering SAU-positive and NAS-positive milk separately, the number of differential proteins was higher in the former group: 162 in SAU-positive milk (128 increased and 34 decreased) and 127 in NAS-positive milk (108 increased and 19 decreased). Of these, 45 proteins were significantly changed only in SAU-positive milk (Table 3, superscript a) and 11 only in NAS-positive milk (Table 3, superscript b).

**Table 3:** Significantly differential proteins in Staphylococcus-positive milk with R_SC_ ≥ 1.5 or R_SC_ ≤ −1.5.

Accession	Description	R_SC_ Pos/Neg	R_SC_ SAU/Neg	R_SC_ NAS/Neg	General function
P48616	Vimentin*	4.78	4.96	4.58	S
A0A0A7NSG7	Cathelicidin 4*	4.75	4.72	4.78	I
Q1JPB0	Leukocyte elastase inhibitor*	4.09	4.17	4	I
X5I0G0	Cathelicidin 1*	4.04	4.1	3.98	I
C8CF42	Probactenecin 7*	3.91	4.02	3.78	I
P46193	Annexin A1	3.86	4.02	3.67	I
P52176	Matrix metalloproteinase-9*	3.8	3.93	3.66	I
P54228	Cathelicidin 6*	3.67	3.72	3.62	I
A0A0A7V3V9	Cathelicidin 2*	3.64	3.74	3.52	I
E1AHZ7	Protein S100A8*	3.52	3.54	3.51	I
Q92176	Coronin-1A*	3.51	3.51	3.51	I
A5D7D1	Alpha-actinin-4	3.48	3.63	3.31	S
P62803	Histone H4*	3.47	3.67	3.24	S
Q2KJD0	Tubulin beta-5 chain	3.45	3.63	3.25	S
Q0VCM4	Glycogen phosphorylase	3.42	3.43	3.42	CM
Q3MHM5	Tubulin beta-4B chain	3.37	3.49	3.23	S
Q3T149	Heat shock protein beta-1*	3.33	3.39	3.26	PD
Q27991	Myosin-10*	3.31	3.28	3.34	S
L0L830	Cathelicidin 7*	3.26	3.37	3.15	I
P40673	High mobility group protein B2*	3.25	3.43	3.04	I
Q5MAR3	Integrin beta*	3.22	3.48	2.9	I
Q0VCW4	L-serine dehydratase/L-threonine deaminase*	3.09	3.16	3.01	AM
P55052	Fatty acid-binding protein, epidermal*	3.08	3.08	3.08	LM
P02253	Histone H1.2*	3.08	3.06	3.11	S
F6MFD5	Peptidoglycan-recognition protein	3.08	3.26	2.86	I
P62808	Histone H2B type 1	3.04	3.04	3.04	S
Q9TU25	Ras-related C3 botulinum toxin substrate 2	2.97	2.99	2.95	I
Q9TU03	Rho GDP-dissociation inhibitor 2*	2.97	3.13	2.79	S
Q2TBL6	Transaldolase*	2.96	3.05	2.86	CM
V9LY96	Cathelicidin 5*	2.95	3.05	2.84	I
Q2HJ86	Tubulin alpha-1D chain	2.95	3.11	2.76	S
Q3SWX7	Annexin A3*	2.86	3.04	2.65	I
Q3B7N2	Alpha-actinin-1	2.85	3.01	2.68	S
Q5E9F5	Transgelin-2*	2.85	2.83	2.86	S
Q3SYV4	Adenylyl cyclase-associated protein 1	2.84	2.78	2.9	S
Q2KJH4	WD repeat-containing protein 1*	2.81	2.63	2.97	S
P68432	Histone H3.1*	2.79	2.91	2.66	S
A7E3Q8	Plastin-3*	2.79	2.86	2.7	S
P81948	Tubulin alpha-4A chain	2.74	2.93	2.51	S
Q3MHR7	Actin-related protein 2/3 complex subunit 2*	2.68	2.61	2.75	S
A7MB62	Actin-related protein 2	2.62	2.6	2.64	S
Q5E9B1	L-lactate dehydrogenase B chain	2.61	2.69	2.52	CM
M1JVB9	Haptoglobin	2.59	2.68	2.49	I
A0A0C5AGQ3	Lysozyme*	2.59	2.64	2.55	I
Q6B855	Transketolase	2.59	2.65	2.54	CM
P02584	Profilin-1	2.58	2.6	2.56	S
Q5E956	Triosephosphate isomerase	2.53	2.56	2.49	S
P10103	High mobility group protein B1	2.52	2.73	2.28	I
A2I7N1	Serpin A3-5	2.52	2.67	2.35	PD
Q3ZBD7	Glucose-6-phosphate isomerase	2.5	2.59	2.4	CM
P0C0S9	Histone H2A type 1	2.5	2.54	2.45	S
Q3T0P6	Phosphoglycerate kinase 1	2.5	2.53	2.47	CM
P19858	L-lactate dehydrogenase A chain	2.48	2.43	2.52	CM
Q3T114	Ribonuclease UK114*	2.47	2.4	2.53	CP
Q32LE5	Isoaspartyl peptidase/L-asparaginase*	2.46	2.34	2.58	CP
P30922	Chitinase-3-like protein 1	2.45	2.32	2.57	I
Q2HJ60	Heterogeneous nuclear ribonucleoproteins A2/B1*	2.39	2.66	2.06	NM
A5A4L2	Histone H2B*^,a^	2.38	2.59		I
P79136	F-actin-capping protein subunit beta	2.32	2.41	2.23	S
P61157	Actin-related protein 3	2.31	2.27	2.35	S
Q5KR47	Tropomyosin alpha-3 chain	2.31	2.37	2.24	S
P09867	Heterogeneous nuclear ribonucleoprotein A1*	2.3	2.45	2.14	S
A4IF97	Myosin regulatory light chain 12B*	2.27	2.39	2.14	S
A3KMV5	Ubiquitin-like modifier-activating enzyme 1*	2.26	2.57	1.87	PD
Q2HJ57	Coactosin-like protein*	2.22	2.24	2.21	I
Q58CQ2	Actin-related protein 2/3 complex subunit 1B*	2.2	2.22	2.18	S
G3N131	Histone H1.1*	2.19			S
P49951	Clathrin heavy chain 1*	2.08	2.22	1.92	S
A5D7A0	EF-hand domain-containing protein D2*	2.08	2.2	1.95	S
Q2TA49	Vasodilator-stimulated phosphoprotein	2.06	2.42	1.58	S
Q3ZC84	Cytosolic non-specific dipeptidase	2.05	2.18	1.91	OM
P10096	Glyceraldehyde-3-phosphate dehydrogenase	2.05	2.14	1.97	CM
P60661	Myosin light polypeptide 6	2.02	2.15	1.87	S
Q3T0D0	Heterogeneous nuclear ribonucleoprotein K*	2.01	2.08	1.94	NM
P33433	Histidine-rich glycoprotein^a^	2.01	2.52		I
Q4U5R3	Proteasome activator complex subunit 1	2.01	2.07	1.96	PD
Q32PI5	Serine/threonine-protein phosphatase 2A 65 kDa regulatory subunit A alpha isoform	1.98	2.29	1.59	S
Q56JZ9	Glia maturation factor gamma*	1.97	2.02	1.92	S
Q0VCX1	Complement C1s subcomponent*	1.96	2.24	1.62	I
Q3SZH7	Leukotriene A-4 hydrolase*	1.96	1.91	2.02	I
P31081	60 kDa heat shock protein, mitochondrial*	1.95	2.12	1.76	I
Q32LP0	Fermitin family homolog 3*	1.94	2.15	1.69	I
D2U6Q0	Serum amyloid A protein*	1.93	1.86	2	I
P62326	Thymosin beta-4*	1.91	2.03	1.78	I
P13752	BOLA class I histocompatibility antigen, alpha chain BL3-6*	1.9	2.08	1.7	I
Q2KJI3	Protein FAM49B*	1.89	1.87	1.92	I
Q762I5	Resistin*	1.89	1.92	1.85	LM
Q76LV2	Heat shock protein HSP 90-alpha	1.88	2.06	1.68	PD
Q3SZ54	Eukaryotic initiation factor 4A-I	1.85	1.82	1.88	GE
Q3MHL4	Adenosylhomocysteinase	1.83	1.82	1.84	AM
P80724	Brain acid soluble protein 1*	1.82	1.61	2	GE
P60712	Actin, cytoplasmic 1	1.8	2.01	1.57	S
J9Q6V1	Glutathione peroxidase*	1.8	2.05	1.5	OM
P55859	Purine nucleoside phosphorylase*	1.78	1.51	2	NM
Q9XSJ4	Alpha-enolase	1.77	1.8	1.75	CM
Q27970	Calpain-1 catalytic subunit^a^	1.77	2.13		PD
Q865V6	Macrophage-capping protein*	1.76	1.65	1.86	S
Q9GMB8	Serine–tRNA ligase, cytoplasmic^a^	1.74	2.02		AM
Q9BGI1	Peroxiredoxin-5, mitochondrial	1.73	1.92	1.52	OM
P51122	Acidic leucine-rich nuclear phosphoprotein 32 family member A^a^		1.72		CT
Q148J6	Actin-related protein 2/3 complex subunit 4*	1.7	1.71	1.69	S
A4FUA8	F-actin-capping protein subunit alpha-1*	1.68	1.63	1.73	S
P06868	Plasminogen	1.68	1.77	1.58	C
P07589	Fibronectin	1.67			I
P13753	BOLA class I histocompatibility antigen, alpha chain BL3-7*^,a^	1.65	1.96		I
O46522	Cytochrome b-245 heavy chain^a^	1.64	2		I
Q3SZI4	14-3-3 protein theta	1.62	1.69	1.55	GE
A6H742	Plastin-1*	1.62	1.54	1.69	S
D2U6Z5	Ceruloplasmin^b^	1.61		1.75	IM
Q3T035	Actin-related protein 2/3 complex subunit 3*^,a^	1.57	1.71		S
P20000	Aldehyde dehydrogenase, mitochondrial*^,a^	1.57	1.71		CM
P01030	Complement C4^b^	1.57		1.72	I
P0C0S4	Histone H2A.Z^a^	1.56	1.66		S
Q27996	Lysozyme C, tracheal isozyme	1.56	1.51	1.61	I
Q5E9B7	Chloride intracellular channel protein 1	1.55	1.52	1.57	C
P68138	Actin, alpha skeletal muscle^a^	1.54	1.72		S
X2IZ01	Signal transducer and activator of transcription^a^	1.54	1.59		I
Q3ZBT1	Transitional endoplasmic reticulum ATPase^a^	1.54	1.68		CT
Q2HJG5	Vacuolar protein sorting-associated protein 35^a^	1.54	1.84		I
O97680	Thioredoxin^a^	1.53	1.79		OM
P19483	ATP synthase subunit alpha, mitochondrial^b^			1.52	CT
P02676	Fibrinogen beta chain^a^		1.79		C
P02672	Fibrinogen alpha chain^a^		1.83		I
Q5E9R3	EH domain-containing protein 1^a^		1.78		CaM
P00829	ATP synthase subunit beta, mitochondrial^a^		1.66		OM
P81644	Apolipoprotein A-II^a^		1.64		LM
Q3B7M5	LIM and SH3 domain protein 1^a^		1.61		S
A6H7G2	Drebrin-like protein^a^		1.58		I
Q3ZBH0	T-complex protein 1 subunit beta^a^		1.57		PD
Q3MHL7	T-complex protein 1 subunit zeta^a^		1.52		PD
Q7SIH1	Alpha-2-macroglobulin^a^		1.52		I
P27214	Annexin A11^a^		1.52		S
Q3SZV7	Hemopexin^a^		1.5		IM
P11116	Galectin-1^b^			1.61	I
P35466	Protein S100-A4^b^			1.6	I
O02675	Dihydropyrimidinase-related protein 2*^,b^			1.51	S
Q3T054	GTP-binding nuclear protein Ran^b^			1.61	CT
Q3T0D7	GTP-binding protein SAR1a	−1.51			CT
Q71SP7	Fatty acid synthase^a^	−1.52	−1.8		LM
P02638	Protein S100-B	−1.53			I
P10790	Fatty acid-binding protein, heart^a^		−1.53		LM
M9WP41	BTN1A1^a^		−1.57		S
Q17QE5	Calcium and integrin-binding protein 1^a^	−1.56	−1.85		I
Q9N0T1	Stanniocalcin-1^a^	−1.57	−1.9		CaM
A0A0C5GDU2	Myostatin^a^		−1.59		S
Q6RUS0	Butyrophilin^b^	−1.6		−1.75	LM
G1AQP3	Xanthine dehydrogenase/oxidase^a^	−1.61	−1.81		LM
E9NV81	ATP-binding cassette sub-family G (WHITE) member 2^a^	−1.62	−1.95		LM
Q05927	5′-nucleotidase^b^	−1.65		−1.88	NM
M4QG36	Insulin induced protein 1^a^	−1.65	−1.88		S
O77588	Procollagen-lysine, 2-oxoglutarate 5-dioxygenase 1^a^	−1.68	−2.19		S
A5PK13	Volume-regulated anion channel subunit LRRC8C	−1.68			LM
Q2KIY5	Putative phospholipase B-like 2^a^	−1.69	−2.24		LM
P81127	Gamma-soluble NSF attachment protein	−1.71			CT
Q58CY6	Prostamide/prostaglandin F synthase^b^	−1.73		−1.83	LM
Q06805	Tyrosine-protein kinase receptor Tie-1	−1.73			A
P37980	Inorganic pyrophosphatase	−1.74	−1.99	−1.53	LM
Q3T0Q2	Transmembrane protein 59	−1.75	−1.87	−1.63	CT
Q3SYV1	Transmembrane protein 263	−1.77			CT
Q3SYS6	Calcineurin B homologous protein 1	−1.81	−1.87	−1.75	CaM
Q58DD4	Syndecan-2^a^	−1.81	−2.13		S
D3K0R6	Plasma membrane calcium-transporting ATPase 4	−1.93	−2.04	−1.82	CaM
C1KGU3	Solute carrier family 11 member 2	−1.93	−2.02	−1.85	CT
Q5GJ77	Glycerol-3-phosphate acyltransferase 1, mitochondrial	−2.01	−2.13	−1.91	LM
A0A088F8E5	Acyl-CoA synthetase short-chain family member 2	−2.08	−2.19	−1.97	LM
P08239	Guanine nucleotide-binding protein G(o) subunit alpha	−2.14	−2.25	−2.03	CT
P08169	Cation-independent mannose-6-phosphate receptor	−2.15	−2.47	−1.88	I
Q3ZBE9	Sterol-4-alpha-carboxylate 3-dehydrogenase, decarboxylating	−2.17	−2.65	−1.8	LM
Q2YDI9	Ferritin, mitochondrial	−2.28	−2.4	−2.17	IM
Q58DW6	Ras-related protein Rab-25	−2.28	−2.39	−2.17	CR
Q3MHW6	Monocarboxylate transporter 1	−2.48	−2.55	−2.41	CM
A0A097P9M4	Long-chain fatty acid-CoA ligase 1	−2.6	−3.2	−2.17	LM
P84466	Lanosterol synthase	−2.71	−2.83	−2.61	LM
Q3MHX6	Protein OS-9^a^		−1.57		S
Q2TBX4	Heat shock 70 kDa protein 13^a^		−1.59		PD
Q0VD19	Sphingomyelin phosphodiesterase^a^		−1.72		LM
Q1RMU3	Prolyl 4-hydroxylase subunit alpha-1^a^		−1.73		S
Q5EA88	Glycerol-3-phosphate dehydrogenase [NAD(+)], cytoplasmic^a^		−1.8		CM
Q0IIG7	Ras-related protein Rab-5A^a^		−2.11		CT
A6QR11	Protein kinase C-binding protein NELL2^a^		−2.37		CaM
Q5E9B5	Actin, gamma-enteric smooth muscle^b^			−3.76	S

The table reports the differential proteins obtained when considering all staphylococcus-positive milk samples (Pos/Neg), only SAU-positive milk samples (SAU/Neg) or only NAS-positive milk samples (NAS/Neg). The general functional classification is indicated as follows: A, angiogenesis; AM: aminoacid metabolism; CaM, calcium metabolism; CM, carbohydrate metabolism; CP: catabolic process; CT, cellular transport; C, coagulation; GE: gene expression; I, immunity; IM, iron metabolism; LM: lipid metabolism; NM: nucleotide metabolism; OM: oxidative metabolism; PD, protein degradation; S, structure. Gene ontology results are detailed in Supplementary File 1.

*Detected in staphylococcus-positive milk (≥2 peptide spectrum matches, PSM) and not detected in healthy milk. ^a^Significantly changed only in SAU-positive milk. ^b^Significantly changed only in NAS-positive milk.

**Figure 2:**
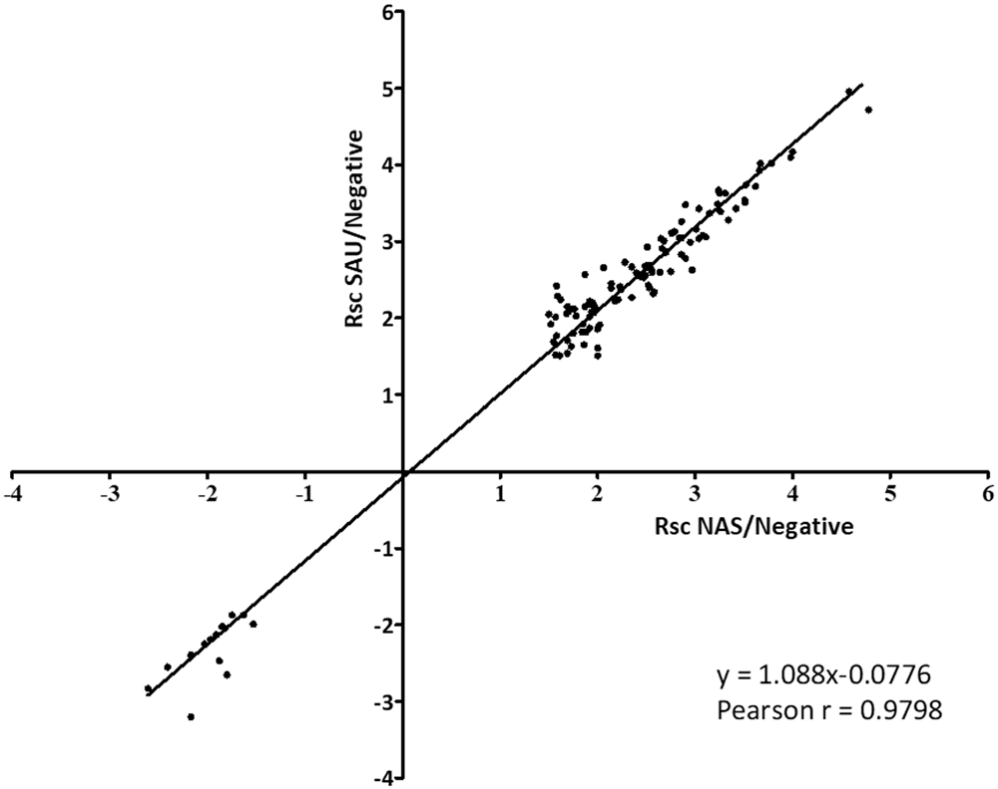
Correlation between the abundance of differential proteins in SAU-positive and NAS-positive samples. The scatter plot illustrates the correlation existing between common proteins increased in milk of buffaloes with subclinical mastitis due to SAU or NAS IMI and highlights the higher intensity of changes in SAU IMI (slope >1, *p* < 0.0005). X axis: R_SC_ values measured when considering only NAS IMI. Y axis: R_SC_ values measured when considering only SAU IMI. Only common proteins with R_SC_ ≥ 1.5 or R_SC_ ≤ −1.5 and p ≤ 0.05 are reported in the plot.

Protein abundance changes were generally in agreement (Pearson r = 0.9798) and were typically more intense in SAU-positive milk than in NAS-positive milk, as demonstrated by the Wilcoxon test (*p* value < 0.0005, Supplementary File, Sheet 6) and visualized by the scatter plot in Fig. 2 (slope 1.088).

### Functional characterisation of differential proteins

**Figure 3:**
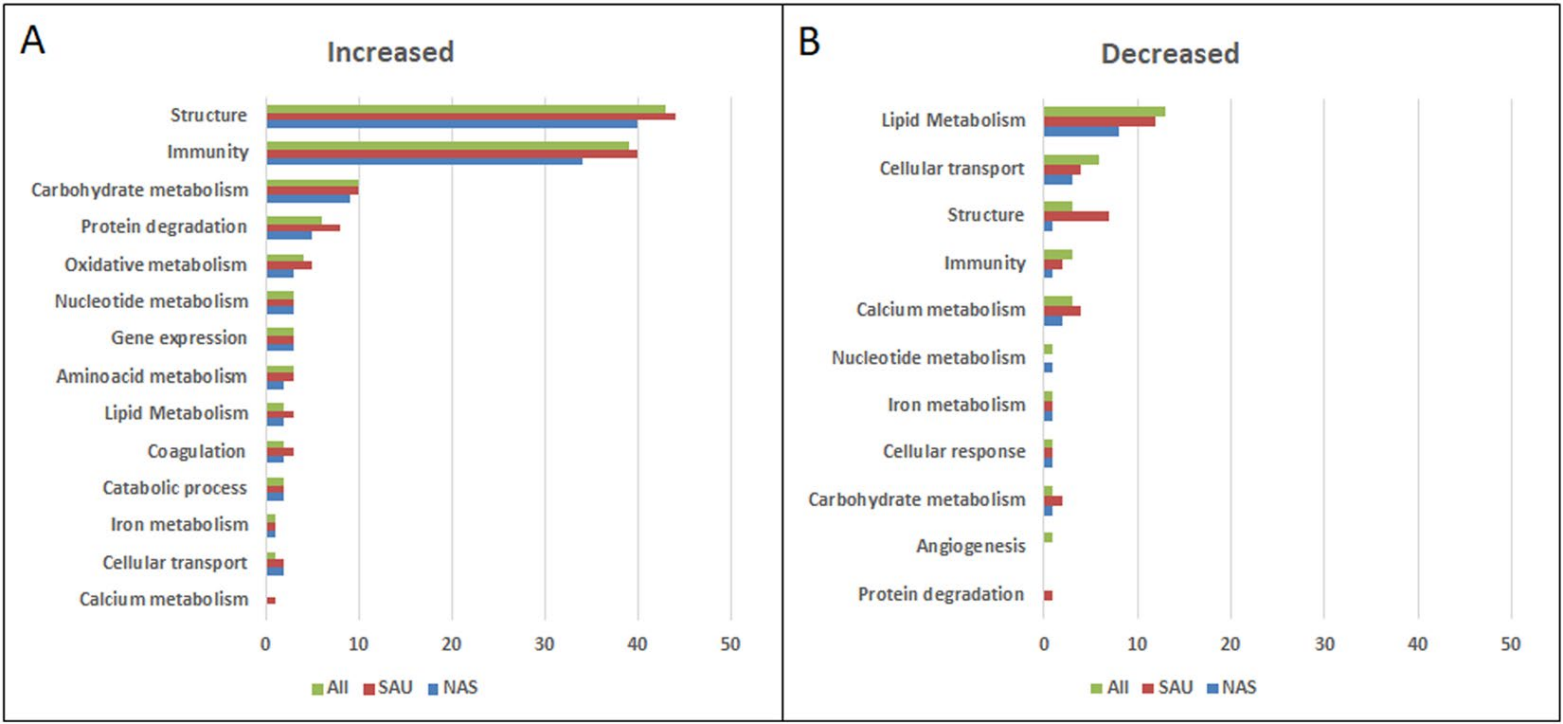
Function distribution of proteins increased and decreased in staphylococcus-positive milk when compared to healthy milk. The number of proteins belonging to each function is indicated. Green: all staphylococcus-positive samples; Red: SAU-positive samples; Blue: NAS-positive samples.

The biological functions affected by staphylococcal mastitis were investigated by means of gene ontology and functional analysis. (Supplementary File 1, Sheets 7–10). Results are detailed in Table 3 and summarised in Fig. 3.

#### Increased proteins

Results are summarised in Fig. 3A. When considering proteins increased in all staphylococcus-positive milk samples (n = 152), the highest number of proteins had structural functions (n = 43, 28.29%), including actin and actin-binding proteins, tubulins, and other cytoskeletal proteins. Histones were comprised in this ontology class because of their structural function in nucleosomes; nevertheless, histones also play a significant role in innate immunity of the mammary gland within Neutrophil Extracellular Traps (NETs)^19,20^. Immunity was the function with the second higher number of proteins (n = 39, 25.66%). Cathelicidins were the most significantly increased protein family, with high R_SC_ values: cathelicidin 4 (R_SC_ 4.75), cathelicidin 1 (R_SC_ 4.04), probactenecin 7 (R_SC_ 3.91), cathelicidin 6 (R_SC_ 3.67), cathelicidin 2 (R_SC_ 3.64), cathelicidin 7 (R_SC_ 3.26), and cathelicidin 5 (R_SC_ 2.95). This class also included antimicrobial proteins and neutrophil granule proteins such as S100 proteins, leukocyte elastase inhibitor, matrix metalloproteinase, and two lysozyme proteoforms (R_SC_ 2.59 and R_SC_ 1.56, respectively). The acute phase proteins haptoglobin (R_SC_ 2.59) and serum amyloid (R_SC_ 1.93) were also significantly increased. Other proteins of interest were high mobility group protein B2 (R_SC_ 3.25), epidermal fatty-acid binding protein (R_SC_ 3.08), peptidoglycan-recognition protein (R_SC_ 3.08), and complement fragments. In line with its antimicrobial function, Histone H2B (R_SC_ 2.38) was included in this ontology class. Other significantly increased proteins belonged to carbohydrate metabolism (n = 10) followed by protein degradation (n = 6), and oxidative metabolism (n = 4). Aminoacid metabolism (n = 3), gene expression and nucleotide metabolism ensued (n = 3). Proteins involved in catabolic process, lipid metabolism, coagulation (n = 2), cellular transport, and iron metabolism (n = 1) were also represented. When considering only SAU-positive milk, some functions were represented by a higher number of significant proteins: structure (44 *vs* 43 proteins), immunity (40 *vs* 39), protein degradation (8 *vs* 6), oxidative metabolism (5 *vs* 4), lipid metabolism, coagulation (3 *vs* 2), and cellular transport (2 *vs* 1). Calcium metabolism was also highlighted (n = 1). On the other hand, when considering only NAS-positive milk, less significant proteins were generally observed in most classes.

#### Increased networks

**Figure 4:**
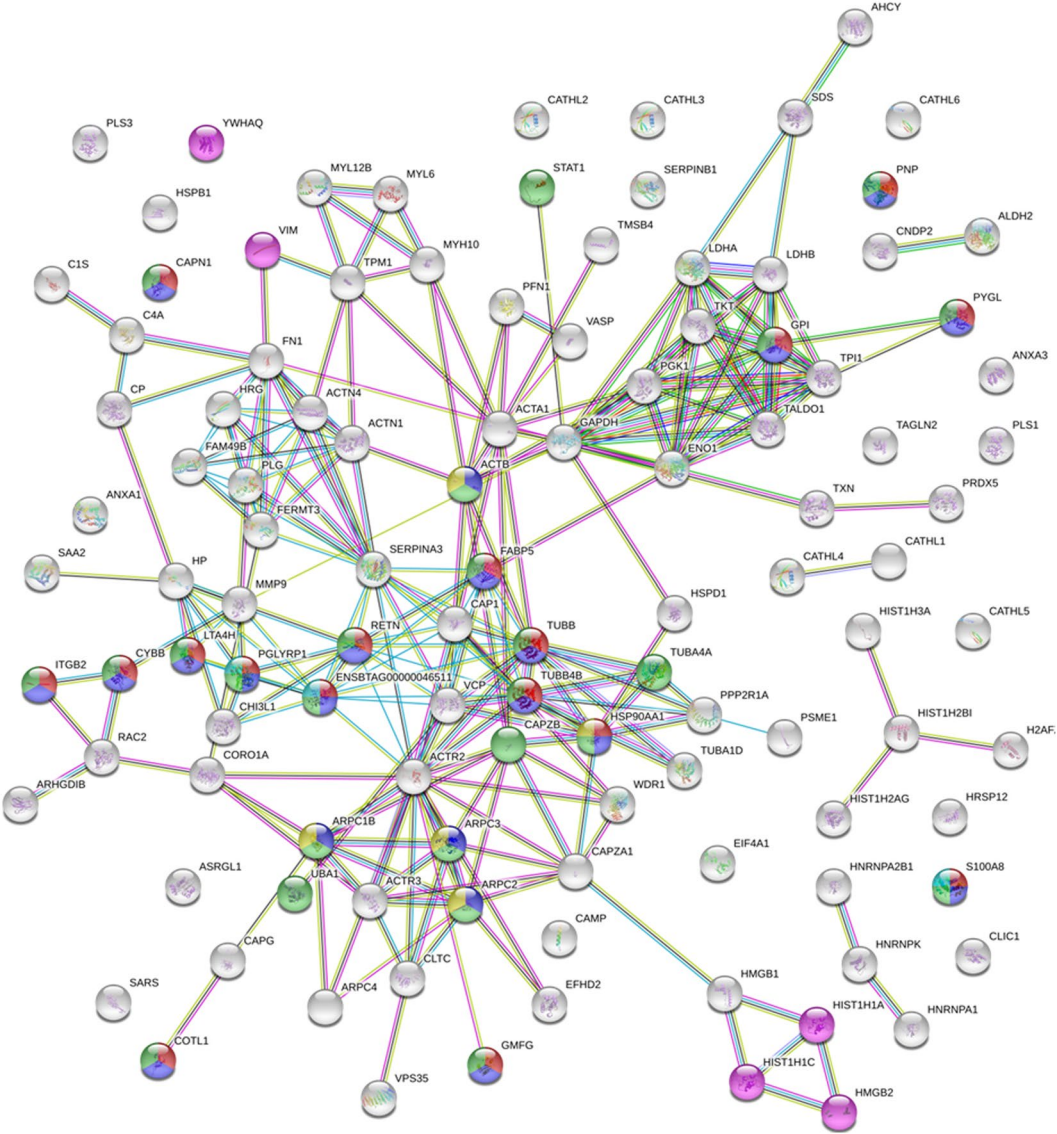
Reactome network according to STRING. Proteins associated with Neutrophil degranulation, Innate Immune System, Immune System, Regulation of actin dynamics for phagocytic cup formation, and Antimicrobial peptides, are indicated in red, pink, green, yellow, and cyan, respectively. Seven different coloured lines link nodes and represent seven types of evidence used in predicting associations. Green lines: neighbourhood evidence; red lines: presence of fusion evidence; blue lines: co-occurrence evidence; black lines: co-expression evidence; purple lines: experimental evidence; light blue lines: database evidence; yellow lines: text-mining evidence.

Based on STRING analysis, the biological process involving most increased proteins was Immunity, with a total of 50 significant term descriptions, ranging from defence response (24 gene counts, FDR < 0.00000005) and response to external stimulus (23 gene counts, FDR < 0.00000005) to lymphocyte activation (3 gene counts, FDR < 0.05). The second process was Structure, with a total of 22 significant term descriptions, ranging from cytoskeleton organization (27 gene counts, FDR < 0.00000005) and actin-filament based process (22 gene counts, FDR < 0.00000005) to actin filament-based movement (FDR < 0.05). Other significant biological processes were Catabolic process, Gene expression, Protein degradation, Carbohydrate metabolism, Oxidative metabolism, Nucleotide metabolism, Aminoacid metabolism, and Coagulation (with 12, 4, 4, 5, 3, 2, 2, and 1 significant term descriptions, respectively). Details are reported in Supplementary File, Sheet 8. Several significant Reactome terms were also obtained for increased proteins. Of note, Neutrophil Degranulation was the most significantl (FDR < 0.00000005) with 17 gene counts, followed by Innate Immune System (FDR < 0.0000005) with 21 gene counts. Immune System, Regulation of actin dynamics for phagocytic cup formation, Apoptosis, and Antimicrobial peptides were other significantly relevant terms (Supplementary Material, Sheet 9). Figure 4 illustrates the Reactome network generated by STRING when investigating the interactions among proteins increased in milk upon staphylococcal mastitis.

#### Decreased proteins

Results are summarised in Fig. 3B. When considering all staphylococcus-positive samples, most of the 22 differential proteins (59.10%) belonged to lipid metabolism (n = 13), followed by cellular transport (n = 6), immunity, structure, and calcium metabolism (n = 3). Other decreased proteins had functions ranging from angiogenesis to cellular response and nucleotide, iron, and carbohydrate metabolism (n = 1). Once again, most protein functions were more represented in SAU-positive samples.

#### Decreased networks

Based on STRING analysis, the biological process involving most decreased proteins was Structure, with a total of 6 significant term descriptions, ranging from anatomical structure morphogenesis (FDR 0.00051) with 7 observed gene counts, to membrane organization (FDR 0.0243) with 3 observed gene counts. Structure was followed by Cellular transport and by Immunity, Lipid metabolism, Cellular homeostasis, Cellular response, Angiogenesis and Nucleotide metabolism. Details are reported in Supplementary File, Sheet 10.

### Western immunoblotting validation

**Figure 5:**
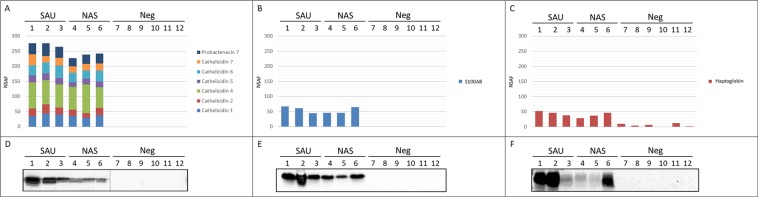
Western immunoblotting validation. Top. Distribution of normalised spectral abundance factor (NSAF) values measured in each sample by shotgun proteomics for (**A**) cathelicidin proteoforms, (**B**) S100A8, and (**C**) haptoglobin. Bottom. Western immunoblotting reactivity of the same samples with (**D**) anti-pan-cathelicidin, (**E**) anti-S100A8, and (**F**) anti-haptoglobin antibodies. SAU: milk samples positive for *Staphylococcus aureus*. NAS: milk samples positive for non-aureus staphylococci. Neg: culture-negative milk. Sample numbers correspond to those listed in Table 1. One microliter of milk was loaded in each lane. Images were cropped to report relevant information. The original experiment images are reported in Supplementary Fig. 1.

According to label-free quantitation, cathelicidins were the most increased protein family upon staphylococcal mastitis, with similar increase in both SAU-positive and NAS-positive milk (Table 3). Other proteins of interest were S100 proteins and acute phase proteins, including haptoglobin, also in view of the previous results generated by proteomic studies carried out on milk from sheep^12,13^ and cows^11,21^ with mastitis. Therefore, these were further investigated by Western Immunoblotting (Fig. 5). Concerning cathelicidin, the abundance of all proteoforms in terms of NSAF values was generally higher in SAU-positive milk than NAS-positive milk, while none was detectable in culture-negative milk (Fig. 5A). Western immunoblotting with anti-pan-cathelicidin antibodies^22,23^ confirmed the shotgun proteomic results; all staphylococcus-positive milk samples were positive for cathelicidins and all healthy milk samples were negative. In addition, a stronger cathelicidin signal was observed in SAU-positive milk (Fig. 5D and Supplementary Fig. 1). S100A8 was also among the top 10 increased proteins in both SAU and NAS IMI, with similar increases in the two milk sample groups (Fig. 5B). Western immunoblotting produced matched results with similar band intensities, with slightly stronger signals in samples with higher NSAF values (Fig. 5E). Haptoglobin was also increased in both sample groups (Table 3), and western immunoblotting confirmed the shotgun proteomics findings. However, some differences in signal intensity were observed, not related to the IMI agent (Fig. 5F). Although few peptides were detected in 4 out of 6 samples by shotgun proteomics, haptoglobin was not detected by western immunoblotting in bacteriologically negative, low SCC quarters (Fig. 5C).

## Discussion

This was the first differential proteomic study investigating the changes induced by infectious mastitis in water buffalo milk. The application of proteomics in this field presents some challenges, since the knowledge regarding buffalo udder health is less well defined when compared to dairy cows and information on sequence and function databases is less complete. Despite these limitations, shotgun proteomics enabled a profound characterisation of buffalo milk proteins, defined the changes that occur in staphylococcal mastitis, and provided indications on their differences in mastitis due to SAU or NAS IMI. Staphylococcal IMI, both by SAU and NAS, induced significant changes even in subclinical conditions; in SAU-positive buffaloes, as expected, these changes were more intense. This could be already appreciated by examining the SDS-PAGE profile; the typical milk pattern was maintained, but the main bands changed in abundance and other bands appeared in the lower molecular weight region, with more intense alterations appearing in SAU-positive milk.

Based on shotgun proteomics, the most significantly increased proteins were of structural origin, followed by immunity, and STRING analysis highlighted Immunity as the most relevant biological process influenced by staphylococcal IMI, followed by structure. This was consistent with the extensive cytoskeletal rearrangements occurring in the mammary epithelium as a result of inflammation, as well as of neutrophil degranulation, chemotaxis, and extravasation; accordingly, Neutrophil Degranulation was the most significantly increased reaction in the Reactome database.

When considering individual proteins, the highest increase in staphylococcal mastitis was observed for vimentin. Vimentin is the most abundant intermediate filament protein with a critical role in stabilisation of cellular architecture^24^. However, recent studies highlighted its involvement in the innate immune response to bacterial pathogens as a ligand for pattern recognition receptors^25^ and as an interactor with NLRP3 for regulation of inflammasome activity^26^. Interestingly, in a recent study on response of bovines to intramammary infection by *Streptococcus uberis*, vimentin was one of the top 15 up-regulated proteins at 57, 81, and 312 hours after intramammary challenge^11^. In view of these results, it will be interesting to further investigate on the role of vimentin in mastitis, as already done in other inflammatory conditions^27^.

When considering protein families, cathelicidins showed the highest increase in staphylococcal mastitis. Seven cathelicidin members were identified, all of them with high R_SC_. Cathelicidins are a family of proteins involved in antimicrobial defence and regulation of immunity that have undergone gene duplication and divergence in ruminants, leading to a family of proteins with similar functions^28,29^. Their significant increase in milk following mammary gland infection has already been reported for cows and ewes, both in natural and experimental infections^11–13,30,31^. Cathelicidins are released by epithelial cells upon microbial sensing, by degranulation of neutrophils that enter the mammary gland as a result of an inflammatory stimulus, as well as together with other granule contents within NETs^32,33^. The presence of NETs and their role in the antimicrobial defence of the water buffalo mammary gland is supported also by the significant increase in histones, the basic component of the nucleoproteic web released during NET formation. Based on gene ontology analysis, histones were classified as structural proteins in consideration of their key role in the nucleosome, but their function in mastitis might be more related to immune response; once again, this contributes to the indication of Immunity as the most relevant biological process according to STRING analysis. Together with cathelicidins, other neutrophil granule proteins were significantly increased, including neutrophil elastase and myeloperoxidase, further supporting the extensive contribution of the neutrophil influx into the mammary gland to the changes observed in the milk proteome. In line with this, integrin was also one of the top increased proteins: integrin is crucial for neutrophil extravasation and entrance in the mammary alveolus^34^. Another important group of significantly increased proteins were acute phase proteins (APP), including haptoglobin, serum amyloid protein A, and ceruloplasmin. The APP increase in milk has been reported and is well known in cows and in sheep^11–13,35^. Another protein of interest was epidermal fatty-acid binding protein (FABP5). Among numerous other biological roles, FABPs are involved in inflammation processes regulated by fatty acids through their interaction with peroxidase proliferator-activated receptors (PPARs), and FABP5, adding to keratinocytes in skin epidermis, is widely expressed in immune cells where it regulates immunological functions^36–38^. Of note, numerous increased proteins carried out defence functions and were involved in innate immunity. Another increased class was proteolysis, both due to increased protein turnover following inflammation as well as to the release in milk of numerous host and pathogen proteases.

On the other hand, over half of the proteins that decreased in staphylococcal mastitis were involved in lipid metabolism. Numerous biosynthetic enzymes were affected, including fatty acid synthase, glycerol-3-phosphate acyltransferase, acyl-Co-A synthetase, sterol-4-alpha-carboxylate 3-dehydrogenase, decarboxylating, long-chain fatty acid-CoA ligase, lanosterol synthase, and others. Of note was also the combined decrease of butyrophilin and xanthine dehydrogenase/oxidase (XD/XO), two of the most important structural components of the milk fat globule (MFG)^39–41^. MFGs are secreted by the epithelial cells of the mammary gland starting from intracellular precursors, the secretory granules^42^. These are transported to the cell surface and are pinched off the cell membrane in a process controlled by the interactions between plasma membrane butyrophilin and butyrophilin in the lipid droplet phospholipid monolayer^43^. XD/XO enables a more efficient secretion of MFGs, and plays a crucial role in stabilising the MFG through its interactions with butyrophilin^44,45^. Combined with the decrease in cellular transport proteins, the second in order of abundance, this suggests that cellular secretion functions, including milk fat globule release, are impaired. Accordingly, STRING analysis confirmed an involvement of the biological processes related to anatomical structure morphogenesis, membrane organization, and lipid metabolism. Interestingly, another FABP isoform, fatty-acid binding protein, heart (FABP3), was decreased in staphylococcal mastitis. Although its role in buffaloes still requires investigation^46^, FABP3 has been reported as positively related to sheep, goat and cow milk quality, being involved in lipid droplet synthesis and accumulation^47–49^. Therefore, the proteomic changes induced by staphylococcal mastitis can potentially affect relevant quantitative, qualitative and structural aspects of water buffalo milk that impact sensorial and textural features of the derived dairy products, including the highly valued “mozzarella di bufala”. It will be of interest to further investigate on this aspect with a combined proteomic and lipidomic approach.

Interesting perspectives for mastitis diagnosis and monitoring are also opened by this study. An efficient detection of mastitis episodes in the herd is crucial for controlling intramammary infections and reducing antibiotic use, and therefore markers and methods providing better diagnostic performances are needed. Several differential proteins have potential as mastitis markers, as already assessed in cows and sheep. Of these, cathelicidins, S100 proteins and haptoglobin have shown to possess diagnostic value when implemented in the ELISA format^21–23,50,51^. The western immunoblotting validation of proteomic results encourages their application also in the water buffalo. Other proteins detected in this study have been implemented in ELISAs for mastitis detection in cows, including milk amyloid^21,52^, and might also be worth investigating for their diagnostic potential in buffalo.

Most of the changes induced by staphylococcal mastitis were more intense in SAU IMI than NAS IMI, although the mean SCC value was similar in the two groups. This emerged in all the experiments carried out in this study. By SDS-PAGE analysis, the banding profile was more altered (Fig. 1); shotgun proteomics indicated a higher number of differential proteins (Tables 2 and 3) as well as a slightly stronger impact on R_SC_ values. Finally, western immunoblotting showed more intense cathelicidin-positive bands (Fig. 4). All these results point to a stronger ability of SAU to alter the buffalo milk proteome when compared to NAS, most likely due to its higher virulence. Other known issues of SAU infections are the contagious nature and therefore ability to spread in the herd, not to mention the adverse consequences of toxins that can contaminate dairy products and cause food poisoning in the human consumer. Therefore, SAU should be eliminated from the herd and adequate biosecurity measures should be applied for preventing its entry and spread in the farm. Nevertheless, the results of this study further highlight the relevant impact of mastitis due to NAS IMI on the buffalo milk proteome as well. Further studies will be needed to investigate on the ability of different NAS species to cause milk alterations in this dairy species. Another aspect that will need to be elucidated is the impact on the milk proteome of staphylococcal colonisation without detectable changes in somatic cell counts, also when considering the recent findings on the mammary gland microbiota^53,54^.

In conclusion, this study generated an extensive dataset of buffalo milk proteins, identified the changes induced by staphylococcal mastitis providing novel information on affected functions and proteins, and revealed differences in the intensity of such alterations according to the pathogen, opening novel perspectives for the development of immunoassay-based systems aimed at improving udder health monitoring in this large ruminant.

## Methods

### Animals and milk samples

The study was carried out on 12 quarter milk samples collected from a water buffalo herd in the context of a survey on mammary gland health in Campania (Italy) receiving an institutional approval by the Ethical Animal Care and Use Committee of the University of Naples “Federico II” (No. 2016/0052967)”. All procedures were carried out conforming to the relevant rules and regulations on animal welfare. Before sampling, all animals enrolled were submitted to a clinical examination. The clinical udder health status was characterised according to our previous study^8^, and CMT was performed on milk samples of each quarter. Teats were carefully cleaned and disinfected with disposable towels embedded with chlorhexidine, and the first streams of milk were discarded. Then, approximately 50 mL of milk was collected aseptically from each teat into sterile vials. Samples were brought to the laboratory and stored at 4 °C for a maximum of 24 h until bacteriological assays and SCC enumeration were performed.

### Bacteriological analysis and somatic cell count

Bacteriological analysis was performed according to the National Mastitis Council standards (2017). Ten μl of each milk sample was spread onto blood agar plates (5% defibrinated sheep blood). Plates were incubated aerobically at 37 °C and examined after 24 h. Colonies were provisionally identified based on Gram stain, morphology, and haemolysis patterns, and the number of each colony type was recorded. Only samples with at least five colonies with the same characteristics were considered positive. Representative colonies were then sub-cultured on blood agar plates and incubated again at 37 °C for 24 h to obtain pure cultures. Gram-positive cocci were tested for catalase and coagulase production. Those showing positive reaction to both tests were identified as SAU. Those showing positive reaction for catalase and negative reaction for coagulase tests were classified as NAS. Somatic cell count was determined using an automated counter (Bentley Somacount 150; Bentley Instruments, Chaska, MN).

### Milk sample preparation for proteomic analysis

Milk was thawed at room temperature and centrifuged at 800 × g at 4 °C for 10 min. Fat was removed and the pellet formed by cells and caseins was resuspended. Skim milk was diluted 1:1 with lysis buffer (2% SDS, 0.4% Tween-20, 130 mM DTT, 500 mM Tris-HCl pH 8.8, and plus protease inhibitor cocktail (Sigma-Aldrich, Saint Louis, MO), incubated at 95 °C for 10 min and then sonicated in a refrigerated water bath for 10 min. The suspension was centrifuged at 10.000 × g for 10 min at 4 °C. The extract was checked for quality by SDS-PAGE as described below. For shotgun proteomic analysis, 7 μl of extract were subjected to filter-aided sample preparation (FASP) as described previously^19^. Briefly, protein samples were subjected to reduction, alkylation, and trypsin digestion on Amicon Ultra-0.5 centrifugal filter units with Ultracel-10 membrane (Millipore, Billerica, MA, USA). Peptide concentration of samples was determined by measuring absorbance at 280 nm with a NanoDrop 2000 spectrophotometer (Thermo Scientific, San Jose, CA, USA) using MassPREP *E*. *coli* Digest Standard (Waters, Milford, MA, USA) to create a calibration curve.

### SDS-PAGE and western immunoblotting

SDS-PAGE and Western immunoblotting were carried out on a Criterion™ Cell with AnykD™ Criterion™ TGX™ precast gels and with a Trans Blot® Turbo™ Blotting System (Bio-Rad Laboratories, Hercules, CA, USA) according to the user manual, as detailed previously with minor modifications^13^. Briefly, 2 μl of the above extract, containing proteins from 1 μl of skimmed milk, were mixed with loading buffer, reduced and denatured, loaded into the wells, and subjected to electrophoretic separation. After the run, gels were stained with Coomassie SafeStain™ (Bio-Rad) for protein visualisation or transferred onto nitrocellulose with the Trans Blot® Turbo^TM^. The nitrocellulose was then blocked, incubated with either monoclonal anti-cathelicidin antibodies as previously described^22^, rabbit polyclonal anti-S100A8 prestige antibodies (Sigma-Aldrich), or sheep polyclonal anti-haptoglobin antibodies (Invitrogen, Carlsbad, CA, USA), followed by the appropriate secondary antibodies, developed with a chemiluminescent substrate, and digitalised with a VersaDocMP 4000 System (Bio-Rad), as detailed previously^55,56^.

### Tandem mass spectrometry analysis of peptides

All peptide mixtures were analysed on a Q-Exactive interfaced with an UltiMate 3000 RSLCnanoLC system (Thermo Scientific, San Jose, CA, USA), as described previously^57^. A total of 4 µg of each peptide mixture were concentrated and washed onto a trapping precolumn (Acclaim PepMap C18, 75 µm × 2 cm nanoViper, 3 µm, 100 Å, Thermo Scientific) and fractionated on a C18 RP column (Acclaim PepMap RSLC C18, 75 µm × 50 cm nanoViper, 2 µm, 100 Å, Thermo Scientific) at flow rate of 250 nL/min using a linear gradient of 245 minutes from 5 to 37.5% eluent B (0.1% formic acid in 80% acetonitrile) in eluent A (0.1% formic acid). Fragmentation occurred by Higher Energy Collisional Dissociation (HCD) and nitrogen as the collision gas. Proteome Discoverer (version 1.4; Thermo Scientific) was used for protein identification using Sequest-HT as search engine. Each MS/MS spectrum was analysed as follows. Database: database custom obtained by merging *Bos taurus* and *Bubalus bubalis* databases downloaded from Swiss-Prot and TrEMBL (release 2017_05 and 2016_11, respectively; enzyme: trypsin, with two missed cleavages allowed; precursor mass tolerance: 10 ppm; MS/MS tolerance: 0.02 Da; charge states: +2, +3, and +4; cysteine carbamidomethylation as static modification and methionine oxidation as dynamic modifications. The percolator algorithm was used for protein significance and for peptide validation (false discovery rate, FDR, <0.01%). Peptide and protein grouping according to the Proteome Discoverer’s algorithm were allowed, applying the strict maximum parsimony principle.

### Proteomic data analysis

Protein abundance changes were assessed by the spectral counting (SpC) approach. For proteins having more than one entry, only those with the highest number of unique peptides and SpCs were selected for downstream analyses. Differential analysis was performed on proteins identified in at least two biological replicates and SpC ≥2 (expressed as Peptide Spectrum Matches, PSMs, in Supplementary File) in at least one sample of the experimental group. The normalised spectral abundance factor (NSAF) and the Rsc (that is, the log2 of the protein abundance ratio) were calculated in order to evaluate the relative abundance of single proteins in all samples and the abundance changes of proteins between groups, respectively^58,59^. Statistical significance was assessed by the beta-binomial test with FDR correction according to Benjamini-Hochberg^41^. Only proteins with R_SC_ ≥ 1.5 or ≤−1.5 in mastitis and a p-value ≤ 0.05 were considered for downstream analyses. Gene ontology (GO) analysis on differentially expressed proteins was carried out based on the biological processes and molecular functions reported by UniProtKB database and integrated with a manual curation of the protein list. The same approach was applied to evaluate protein-protein interaction network using the STRING database (Version 11, http://string-db.org/), after replacing *Bubalus bubalis* UniProt IDs with the corresponding *Bos taurus* UniProt IDs, by sequence alignment of identified peptides using Basic Local Alignment Search Tool (BLAST)^60^. In this analysis, only functional interactions with high confidence (combined score >0.7) were evaluated^61^. The Wilcoxon test^62^ was performed to demonstrate a statistically significant differences between SAU/Neg and NAS/Neg Rsc, by using the MedCalc Statistical Software version 18.9 (MedCalc Software bvba, Ostend, Belgium; http://www.medcalc.org; 2018).

## Supplementary information

Supplementary file

Related Manuscript File

## Data Availability

The data have been deposited to the ProteomeXchange with identifier PXD012355.

## References

[1] IDF bulletin - International Dairy Federation. *The world dairy situation 2007. Bulletin No. 423/2007* (2007).

[2] Cagnardi P (2017). Clinical efficacy and pharmacokinetics of meloxicam in Mediterranean buffalo calves (Bubalus bubalis). PLoS One.

[3] Guccione J, Ciaramella P (2017). Mastitis in Mediterranean Buffaloes. J. Dairy Vet. Sci..

[4] Wanasinghe, D. D. Mastitis among buffalos in Sri Lanka. In *Proc. First World Buffalo Congr. Cairo, Egypt*. 1331–1333 (1985).

[5] Guccione J (2014). Short communication: Effects of systemic treatment with penethamate hydriodide on udder health and milk yields in dry primiparous Mediterranean buffaloes (Bubalus bubalis). J. Dairy Sci..

[6] Guccione J (2016). Short communication: Role of Streptococcus pluranimalium in Mediterranean buffaloes (Bubalus bubalis) with different udder health statuses. J. Dairy Sci..

[7] Guccione J (2017). Efficacy of a polyvalent mastitis vaccine against Staphylococcus aureus on a dairy Mediterranean buffalo farm: Results of two clinical field trials. BMC Vet. Res..

[8] Guccione J, Borriello G, Ciaramella P, Di Loria A (2017). Clinical evaluation of poor milking procedures effects on dairy Mediterranean buffaloes udder health. Large Anim. Rev..

[9] Moroni P (2006). Relationships Between Somatic Cell Count and Intramammary Infection in Buffaloes. J. Dairy Sci..

[10] Guccione J (2014). Clinical outcomes and molecular genotyping of Staphylococcus aureus isolated from milk samples of dairy primiparous Mediterranean buffaloes (Bubalus bubalis). J. Dairy Sci..

[11] Mudaliar M (2016). Mastitomics, the integrated omics of bovine milk in an experimental model of *Streptococcus uberis* mastitis: 2. Label-free relative quantitative proteomics. Mol. BioSyst..

[12] Addis MF (2011). Proteomics and pathway analyses of the milk fat globule in sheep naturally infected by Mycoplasma agalactiae provide indications of the *in vivo* response of the mammary epithelium to bacterial infection. Infect. Immun..

[13] Addis MF (2013). Production and release of antimicrobial and immune defense proteins by mammary epithelial cells following Streptococcus uberis infection of sheep. Infect. Immun..

[14] Boehmer JL (2011). Proteomic analyses of host and pathogen responses during bovine mastitis. J. Mammary Gland Biol. Neoplasia.

[15] D’Ambrosio C (2008). A proteomic characterization of water buffalo milk fractions describing PTM of major species and the identification of minor components involved in nutrient delivery and defense against pathogens. Proteomics.

[16] Jena MK (2015). DIGE based proteome analysis of mammary gland tissue in water buffalo (Bubalus bubalis): Lactating vis-a-vis heifer. J. Proteomics.

[17] Santana AM (2018). Reference 1D and 2D electrophoresis maps for potential disease related proteins in milk whey from lactating buffaloes and blood serum from buffalo calves (Water buffalo, Bubalus bubalis). Res. Vet. Sci..

[18] Vizcaíno JA (2016). 2016 update of the PRIDE database and its related tools. Nucleic Acids Res..

[19] Pisanu S (2015). Neutrophil extracellular traps in sheep mastitis. Vet. Res..

[20] Lippolis JD, Reinhardt TA, Goff JP, Horst RL (2006). Neutrophil extracellular trap formation by bovine neutrophils is not inhibited by milk. Vet. Immunol. Immunopathol..

[21] Thomas FC (2015). The major acute phase proteins of bovine milk in a commercial dairy herd. BMC Vet. Res..

[22] Addis MF (2016). Evaluation of milk cathelicidin for detection of dairy sheep mastitis. J. Dairy Sci..

[23] Addis MF (2016). Evaluation of milk cathelicidin for detection of bovine mastitis. J. Dairy Sci..

[24] Eriksson JE (2009). Introducing intermediate filaments: from discovery to disease. J. Clin. Invest..

[25] Mak, T. N. & Brüggemann, H. Vimentin in Bacterial Infections. *Cells* **5** (2016). 10.3390/cells5020018 PMC4931667 27096872

[26] dos Santos G (2015). Vimentin regulates activation of the NLRP3 inflammasome. Nat. Commun..

[27] Mor-Vaknin N (2013). Murine Colitis is Mediated by Vimentin. Sci. Rep..

[28] Kościuczuk EM (2012). Cathelicidins: family of antimicrobial peptides. A review. Mol. Biol. Rep..

[29] Zanetti M (2004). Cathelicidins, multifunctional peptides of the innate immunity. J. Leukoc. Biol..

[30] Cubeddu T (2017). Cathelicidin production and release by mammary epithelial cells during infectious mastitis. Vet. Immunol. Immunopathol..

[31] Smolenski G (2007). Characterisation of host defense proteins in milk using a proteomic approach. J. Proteome Res..

[32] Brinkmann V (2004). Neutrophil extracellular traps kill bacteria. Science.

[33] Brinkmann V, Zychlinsky A (2012). Neutrophil extracellular traps: Is immunity the second function of chromatin?. J. Cell Biol..

[34] Abram CL, Lowell CA (2009). The ins and outs of leukocyte integrin signaling. Annu. Rev. Immunol..

[35] Ceciliani F, Ceron JJ, Eckersall PD, Sauerwein H (2012). Acute phase proteins in ruminants. J. Proteomics.

[36] Zhang Y (2015). Epidermal Fatty Acid Binding Protein Promotes Skin Inflammation Induced by High-Fat Diet. Immunity.

[37] Li B, Reynolds JM, Stout RD, Bernlohr DA, Suttles J (2009). Regulation of Th17 Differentiation by Epidermal Fatty Acid-Binding Protein. J. Immunol..

[38] Zhang Y (2014). Fatty Acid-Binding Protein E-FABP Restricts Tumor Growth by Promoting IFN- Responses in Tumor-Associated Macrophages. Cancer Res..

[39] Affolter M, Grass L, Vanrobaeys F, Casado B, Kussmann M (2010). Qualitative and quantitative profiling of the bovine milk fat globule membrane proteome. J. Proteomics.

[40] Pisanu S (2013). Characterization of size and composition of milk fat globules from Sarda and Saanen dairy goats. Small Rumin. Res..

[41] Pisanu Salvatore, Ghisaura Stefania, Pagnozzi Daniela, Biosa Grazia, Tanca Alessandro, Roggio Tonina, Uzzau Sergio, Addis Maria Filippa (2011). The sheep milk fat globule membrane proteome. Journal of Proteomics.

[42] Heid HW, Keenan TW (2005). Intracellular origin and secretion of milk fat globules. Eur. J. Cell Biol..

[43] Robenek H (2006). Butyrophilin controls milk fat globule secretion. Proc. Natl. Acad. Sci. USA.

[44] Monks J (2016). Xanthine oxidoreductase mediates membrane docking of milk-fat droplets but is not essential for apocrine lipid secretion. J. Physiol..

[45] Jeong J (2009). The PRY/SPRY/B30.2 domain of butyrophilin 1A1 (BTN1A1) binds to xanthine oxidoreductase: implications for the function of BTN1A1 in the mammary gland and other tissues. J. Biol. Chem..

[46] Dubey PK (2016). Identification of polymorphism in fatty acid binding protein 3 (FABP3) gene and its association with milk fat traits in riverine buffalo (Bubalus bubalis). Trop. Anim. Health Prod..

[47] Liang M (2014). Functional analysis of FABP3 in the milk fat synthesis signaling pathway of dairy cow mammary epithelial cells. Vitr. Cell. Dev. Biol. - Anim..

[48] Moioli B, D’Andrea M, Pilla F (2007). Candidate genes affecting sheep and goat milk quality. Small Rumin. Res..

[49] Shi H (2015). Genes regulating lipid and protein metabolism are highly expressed in mammary gland of lactating dairy goats. Funct. Integr. Genomics.

[50] Wheeler TT (2012). Host-defence-related proteins in cows’ milk. Animal.

[51] Wheeler, T. T. *et al*. Innate immune proteins as biomarkers for mastitis and endometritis. In *Proc. ADSS 294–297* (2012).

[52] Singh M (2015). Estimation of acute phase proteins as early biomarkers of buffalo subclinical mastitis. Asian J. Anim. Vet. Adv..

[53] Catozzi C (2017). The microbiota of water buffalo milk during mastitis. PLoS One.

[54] Addis MF (2016). The bovine milk microbiota: insights and perspectives from -omics studies. Mol. Biosyst..

[55] Pisanu S, Cubeddu T, Uzzau S, Rocca S, Addis MF (2017). Proteomic changes in the ileum of sheep infected with Mycobacterium avium subspecies paratuberculosis. Vet. J..

[56] Addis MF (2009). Generation of high-quality protein extracts from formalin-fixed, paraffin-embedded tissues. Proteomics.

[57] Pisanu S, Biosa G, Carcangiu L, Uzzau S, Pagnozzi D (2018). Comparative evaluation of seven commercial products for human serum enrichment/depletion by shotgun proteomics. Talanta.

[58] Old WM (2005). Comparison of label-free methods for quantifying human proteins by shotgun proteomics. Mol. Cell. Proteomics.

[59] Zybailov B (2006). Statistical analysis of membrane proteome expression changes in Saccharomyces cerevisiae. J. Proteome Res..

[60] Szklarczyk D (2019). STRING v11: protein–protein association networks with increased coverage, supporting functional discovery in genome-wide experimental datasets. Nucleic Acids Res..

[61] Campbell J (2016). Large-Scale Profiling of Kinase Dependencies in Cancer Cell Lines. Cell Rep..

[62] Conover, W. J. *Practical nonparametric statistics*. (Wiley, 1999).

