# Supplementary material for: Proteomic changes in the milk of water buffaloes (Bubalus bubalis) with subclinical mastitis due to intramammary infection by Staphylococcus aureus and by non-aureus staphylococci
Source: Sci Rep. 2019 Nov 1;9:15850. doi: 10.1038/s41598-019-52063-2 (PMC6825138; doi:10.1038/s41598-019-52063-2)

Proteomic changes in the milk of water buffaloes (*Bubalus bubalis*) with subclinical mastitis due to intramammary infection by *Staphylococcus aureus* and by non-aureus staphylococci

Salvatore Pisanu[1], Carla Cacciotto[1#a], Daniela Pagnozzi[1], Giulia Maria Grazia Puggioni[1], Sergio Uzzau[1,2], Paolo Ciaramella[3], Jacopo Guccione[3], Martina Penati[4], Claudia Pollera[4], Paolo Moroni[4,5], Valerio Bronzo[4] & Maria Filippa Addis[1,4*]

[1]Porto Conte Ricerche, Alghero, Italy
[2]Dipartimento di Scienze Biomediche, Università degli Studi di Sassari, Sassari, Italy
[3]Dipartimento di Medicina Veterinaria e Produzioni Animali, Università di Napoli Federico II, Naples, Italy
[4]Dipartimento di Medicina Veterinaria, Università degli Studi di Milano, Milan, Italy
[5]Animal Health Diagnostic Center, Cornell University, Ithaca, NY, USA
[#a]Current address: Dipartimento di Medicina Veterinaria, Università degli Studi di Sassari, Sassari, Italy
Correspondence and requests for materials should be addressed to M.F.A. (e-mail: filippa.addis@unimi.it).
Additional corresponding author: D.P. (e-mail: pagnozzi@portocontericerche.it)

Supplementary Figure 1: Original western blotting experiment images used for preparing Figure 4. Left: anti-cathelicidin antibodies; Middle: anti-S100A8 antibodies; Right: anti-haptoglobin antibodies. Sample numbers refer to milk samples listed in Table 1 of the main article.

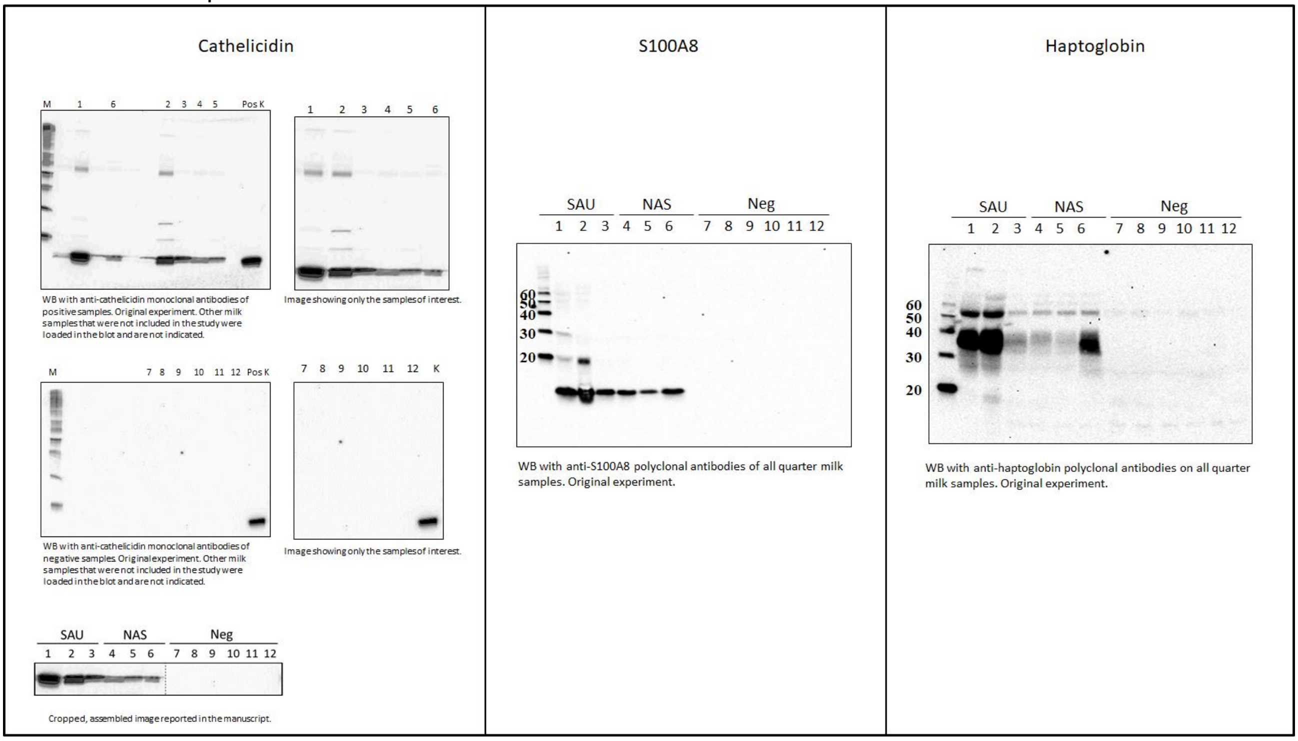

Supplement: Supplementary file 2 — Related Manuscript File [file 41598_2019_52063_MOESM2_ESM.pdf]
